# Context-Dependent Mechanisms of Oncologic Photodynamic Therapy: A Framework Linking Treatment Parameters to Cellular, Vascular, and Immune Responses

**DOI:** 10.3390/ijms27167465

**Published:** 2026-08-20

**Authors:** Xuewu Zhang, Xujia Wang, Chengbo Zhang, Yongmei Hu, Zhangyu Jia, Junyi Li, An Jiang

**Affiliations:** 1 Department of Hepatobiliary Pancreas and Liver Transplantation, The Second Affiliated Hospital of Xi’an Jiaotong University, Xi’an 710004, China; 2 Key Laboratory of Surgical Critical Care and Life Support, Xi’an Jiaotong University, Ministry of Education, Xi’an 710004, China

**Keywords:** photodynamic therapy, photosensitizer localization, drug–light interval, oxygen, dosimetry, vascular-targeted photodynamic therapy, immunogenic cell death, tumor microenvironment, photodetection, critical narrative review

## Abstract

Photodynamic therapy (PDT) combines a photosensitizer (PS), light, and molecular oxygen to generate cytotoxic reactive species, but nominally similar regimens can produce different biological responses because prescribed inputs do not directly specify the biologically active exposure state. Here, this intermediate state is treated as a multidimensional profile defined by PS localization and photoactive availability, the intratissue light field, oxygen dynamics, and vascular conditions at illumination. The relative contributions of direct tumor-cell injury, vascular damage, inflammatory signaling, and adaptive immune modulation also depend on drug–light interval (DLI), treatment sequence, vascular architecture, and tumor–host context. This critical narrative review applies a parameter–exposure state–mechanism–endpoint framework to oncologic PDT. It distinguishes spatially restricted primary photochemical injury from downstream multicellular effects and appraises the evidentiary thresholds for immunogenic cell death (ICD) and systemic immune claims. It also proposes reporting considerations and translational priorities to improve study comparability. PDT mechanisms should be interpreted against measurable treatment parameters rather than presumed from a PS label or prescribed dose alone.

## 1. Introduction

Photodynamic therapy (PDT) uses a photosensitizer (PS), light of a suitable wavelength, and molecular oxygen to generate short-lived oxidizing species in illuminated tissue. Its enduring clinical appeal is the possibility of spatial control: illumination can be confined to a selected field, while PS pharmacokinetics, formulation, and biological distribution create additional layers of selectivity. PDT has consequently been developed across dermatologic, aerodigestive, biliary, urologic, pulmonary, and other oncologic settings, as well as in non-oncologic applications. The treatment is not, however, a uniform modality. Distinct PS classes, routes of administration, drug–light intervals (DLIs), illumination geometries, and tissue optical conditions can produce qualitatively different exposure patterns and biological sequelae [1,2,3].

Three broad routes of tumor control have long been recognized: direct injury to malignant cells, damage to tumor-associated vasculature, and inflammatory or immune-mediated effects. These routes coexist rather than operate as discrete, mutually exclusive programs. A vascularly concentrated PS illuminated at a short DLI may produce vascular shutdown as a major early event, whereas a PS that has redistributed to tumor-cell organelles at a later DLI may favor intracellular oxidative injury. Light delivery and oxygen dynamics can further change the balance even for the same PS and tumor model. For this reason, statements that PDT “works by apoptosis”, “works by vascular occlusion”, or “acts as an in situ vaccine” are often incomplete unless the treatment context and outcome measures are specified [4,5,6,7].

This review adopts a practical interpretive position: PDT mechanisms are best reported as relative contributions conditioned by measurable treatment parameters. A parameter–exposure state–mechanism–endpoint framework provides a structured way to do this while preserving biological heterogeneity and the uncertainty of causal attribution. It can make claims more testable, distinguish observations from plausible interpretation, and keep mechanistic conclusions aligned with the available evidence. The focus is oncologic PDT, with particular attention to the translational interfaces of targeted delivery, photodetection/dosimetry, vascular response, and immune modulation.

Prior PDT reviews have established the importance of cellular, vascular, and immune mechanisms, as well as optical and pharmacological determinants of response. Recent broad syntheses also catalogue molecular responses and recognize the influence of photosensitizer localization, light delivery, oxygen availability, and tumor context [1,3,4,8,9,10]. Other reviews have focused on immune effects, clinical immunoadjuvant development, or personalized dosimetry [11,12,13]. Two recurrent cross-study comparison problems nevertheless remain. Identical prescribed fluence can produce different intratissue oxygen and reacted-dose trajectories when fluence rate, geometry, and perfusion differ; conversely, the same PS can shift from predominantly vascular to tissue or cellular exposure as DLI and formulation change. The present review addresses these problems by formalizing the biologically active exposure state as an intermediate layer between nominal protocol inputs and downstream mechanisms. It then links mechanistic claims to proportionate evidentiary thresholds and provides a common matrix for comparing protocols without treating PS identity or prescribed dose as a sufficient mechanistic descriptor.

To assess whether this reporting gap is observable in recent oncologic PDT reports rather than only conceptual, we conducted a focused abstract-level audit of 30 PubMed-indexed original studies published in 2024–2026 (Supplementary Table S1). At least one intervention-defining PDT input was explicit in all 30 abstracts, whereas an exposure-state-related measurement or surrogate was explicit in 13/30 (43.3%). Explicit mechanistic attribution and a mechanism-matched endpoint were each present in 23/30 (76.7%), and 10/30 reports (33.3%) explicitly contained all four prespecified elements. These proportions describe the audited cross-section rather than the PDT literature as a whole, but they show that incomplete linkage is observable, particularly at the intermediate exposure-state layer.

## 2. Scope and Interpretive Approach

This critical narrative review synthesizes and interprets evidence through an explicit conceptual framework rather than through systematic or quantitative evidence synthesis. The framework was constructed by mapping treatment-parameter domains to measurable exposure-state variables, the relative contributions of cellular, vascular, and immune mechanisms, mechanism-matched endpoints, and clinical interpretation; it was then used as the organizing lens for the literature synthesis. The review was not designed for exhaustive study identification, pooled effect estimation, or formal risk-of-bias assessment, so PRISMA and meta-analytic methods were not applied. Citations within individual subsections are therefore representative rather than exhaustive.

The core domains were selected according to three linked criteria: they represent established determinants of PDT exposure or response; they can alter the compartment, magnitude, or timing of photochemical injury; and they can be connected to at least one measurable or inferable intermediate variable and a mechanism-matched endpoint. Accordingly, the framework integrates protocol determinants—including PS identity and formulation, DLI, light delivery, oxygen state, vascular architecture, treatment sequence, and tumor–host context—with the principal cellular, vascular, and immune response compartments. The framework is deliberately selective: domains are included when they directly improve exposure characterization, cross-study comparison, or calibration of a mechanistic claim [1,3,4,5,8,9].

Established dosimetry estimates delivered photochemical exposure from combinations of light, photosensitizer, oxygen, and reaction-related measurements [8]. The present framework adds an interpretive layer by using the measured or inferred exposure state to connect protocol inputs with the relative cellular, vascular, and immune contributions supported by mechanism-matched endpoints. This distinction also motivates the parameter-specific comparison matrix presented in Section 8.

Here, the biologically active exposure state is defined as a context-specific multidimensional profile of PS localization and photoactive availability, intratissue light delivery, dynamic oxygenation, and relevant vascular conditions at illumination. Selected components can be measured or inferred with complementary optical, dosimetric, imaging, and physiological methods, although complete simultaneous characterization is rarely feasible in patients. The framework uses these components to guide measurement priorities and calibrate mechanistic inference, while established dosimetry remains the appropriate basis for dose estimation and clinical dosing decisions [8,13,14,15].

To improve transparency, targeted PubMed searches were updated through 10 July 2026. The principal search structure was (“photodynamic therapy” OR PDT) combined with one or more terms covering photosensitizer localization or pharmacokinetics, drug–light interval, light delivery or fluence rate, dosimetry, oxygen or hypoxia, vascular targeting or response, regulated cell death or immunogenic cell death, immune response, and clinical studies. Citation chaining from foundational and recent reviews was used to identify representative primary, translational, and clinical studies. We prioritized reports that linked a treatment parameter to a biologically relevant exposure measure, a compartment-specific response, a functional immune endpoint, or a clinically interpretable PDT or vascular-targeted photodynamic therapy (VTP) outcome. Foundational reviews were used to define established concepts, whereas representative primary, translational, and clinical reports were used for parameter-specific inferences. Selection was iterative and interpretive; no pooled estimates or formal risk-of-bias assessment were undertaken.

To provide an empirical check on the rationale for the framework, we also performed a focused descriptive content audit of 30 PubMed-indexed original oncologic PDT reports from 2024–2026. The sample was treated as a structured descriptive cross-section rather than a probability sample, and the PubMed-indexed abstract was the unit of analysis. Each abstract was coded for four prespecified elements: (i) at least one intervention-defining PDT input; (ii) an exposure-state measurement or surrogate within PS localization/photoactive availability, intratissue light or fluence, oxygen dynamics, or vascular/perfusion conditions; (iii) explicit mechanistic attribution; and (iv) a mechanism-matched endpoint. ROS generation alone was not counted as an exposure-state measurement, and tumor size, response rate, or viability alone was not counted as a mechanism-matched endpoint unless explicitly tied to the claimed mechanism. A negative code means only that the element was not explicit in the abstract, not that it was absent from the full study. Study-level coding, PMIDs, and source links are provided in Supplementary Table S1.

Throughout, four distinctions are maintained. First, photochemical capability is distinguished from delivered photochemical dose. Second, initial subcellular or vascular injury is distinguished from delayed tissue-level consequences. Third, biomarker induction is distinguished from functional mechanism. Fourth, preclinical immune observations are distinguished from clinical evidence of durable systemic benefit. These distinctions are especially important in a field in which new formulations may alter biodistribution, optical behavior, and oxygen availability simultaneously, making simple one-variable explanations unreliable [1,8,16].

## 3. The First Determinant: What Photochemical Dose Reaches Which Compartment?

### 3.1. Photochemistry Is Necessary but Not Sufficient to Predict Biological Outcome

Following absorption of light, an excited PS may transfer energy to molecular oxygen, commonly described as Type II photochemistry, or participate in electron-transfer reactions that generate radical species, commonly described as Type I photochemistry. The relative balance is influenced by PS structure, aggregation state, local oxygen tension, redox environment, and accessible substrates. The resulting oxidants have limited diffusion radii in biological systems. Consequently, the initial photochemical lesion is spatially constrained by the local PS distribution and microenvironment rather than defined solely by the incident fluence [5,17,18].

A high singlet-oxygen yield in a homogeneous solution does not, by itself, establish superior biological activity in a tumor. In tissue, scattering and absorption modify fluence with depth; PS concentration varies across malignant cells, endothelium, stroma, and normal tissue; photobleaching changes the active sensitizer pool during illumination; and oxygen can be depleted faster than it is replenished. Thus, a prescribed light dose should not be equated with absorbed or reacted dose. The concept of PDT dose is better viewed as an integrated exposure involving the PS, local fluence rate, oxygen availability, and time [8,14,15].

This perspective also helps explain why nominally similar protocols can be difficult to compare. Two studies may report the same PS dose and J/cm^2^ but use different wavelengths, beam geometries, tissue thicknesses, DLIs, or illumination rates, thereby creating different biologically effective interventions. A mechanism-oriented manuscript should therefore report both the nominal regimen and the plausible exposure state in the relevant compartment.

The expanding PS landscape reinforces this point. Boron-dipyrromethene (BODIPY) derivatives, transition-metal complexes, and newer molecular designs can alter absorption, triplet-state behavior, photostability, and redox reactivity; none of these chemical features alone specifies the biological mechanism in a tumor. Comparative studies should therefore report the relevant photophysics together with aggregation, localization, and tissue exposure rather than treating a molecular class as a mechanistic conclusion [19,20,21,22,23,24,25].

### 3.2. Photosensitizer Localization and DLI Define the Initial Injury Compartment

PS localization is a major determinant of the first structure exposed to oxidative injury. Depending on molecular properties and carrier design, PSs can associate with plasma membranes, lysosomes, mitochondria, endoplasmic reticulum (ER), Golgi apparatus, nuclei, tumor vasculature, extracellular matrix, or multiple compartments. Lipophilicity, charges, aggregation, protein binding, receptor-mediated uptake, and intracellular trafficking can each alter this distribution. It is therefore more defensible to describe localization as an experimentally observed property of a particular PS–formulation–cell system than as a fixed trait of a PS name [5,26].

The physicochemical properties of a PS can substantially bias its intracellular distribution, but they do not uniquely determine organellar localization. Charges, lipophilicity, amphiphilicity, ionization state, aggregation tendency, and carrier-mediated trafficking collectively influence membrane partitioning, endocytic uptake, and subsequent organellar retention. Because PDT-generated reactive species act over short distances, preferential localization near mitochondria, lysosomes, the ER, or the plasma membrane biases which molecular structures are initially oxidized, thereby shaping the downstream cytotoxic response: mitochondrial photodamage can dissipate membrane potential and promote intrinsic apoptotic signaling; lysosomal injury can release cathepsins and secondarily perturb mitochondria; ER-associated photodamage can induce calcium dysregulation and proteostatic stress; and extensive plasma membrane oxidation can rapidly compromise membrane integrity. These relationships should be interpreted as context-dependent tendencies, rather than deterministic structure–organelle rules, because formulation, PS concentration, exposure time, cell type, and intracellular trafficking dynamics can substantially modify the final subcellular distribution [5,6,26].

The DLI provides a clinically accessible, although imperfect, means of modifying the distribution state at illumination. Early illumination may occur when a circulating PS is still enriched in vascular and stromal compartments; later illumination may permit greater cellular uptake or organellar redistribution. The relationship is PS-specific, tissue-specific, and influenced by route and formulation. Nevertheless, the DLI is not only a logistical interval: it is a biological parameter that can alter the dominant injured compartment and the interpretation of efficacy and toxicity. Studies that compare only total dose without documenting DLI may conceal an important source of mechanistic variation [3,5,7].

Targeted delivery strategies sharpen this principle because their mechanistic meaning depends on the exposure state they create. Antibodies, peptides, lipid nanoparticles, polymeric carriers, cell-membrane coatings, metal–organic frameworks, and other platforms may improve tumor accumulation, reduce aggregation, enable image guidance, or alter intracellular routing. Each can also change pharmacokinetics, protein corona formation, clearance, and the ratio of tumor-cell to vascular exposure. Demonstrating higher tumor fluorescence alone is therefore insufficient to assign a specific death pathway or immune effect; that attribution requires evidence of distribution and functional consequence [27,28,29].

Representative pharmacokinetic and localization studies show that DLI should be interpreted in a PS- and tissue-specific manner. For some circulating PSs, short DLIs can favor vascular or stromal exposure, whereas longer intervals can increase cellular uptake; the direction and magnitude of this change also depend on route and formulation. Padeliporfin (WST-11; TOOKAD Soluble) is intentionally used in vascular-targeted protocols, whereas 5-aminolevulinic acid (ALA)-derived protoporphyrin IX (PpIX) follows a distinct prodrug-to-intracellular-porphyrin pathway. These examples illustrate how DLI modifies exposure without supporting a fixed DLI-to-mechanism rule [3,5,7,30,31,32,33].

### 3.3. Spatially Restricted Oxidative Injury Can Produce System-Level Consequences

Short diffusion distances of singlet oxygen and related oxidants mean that primary oxidation is local. However, biological consequences can spread beyond the initially oxidized molecules through calcium flux, mitochondrial signaling, lysosomal leakage, protease activation, damage-associated molecular pattern release, endothelial activation, microvascular thrombosis, inflammatory-cell recruitment, and changes in perfusion. “Local” photochemistry should therefore not be confused with “cell-autonomous” biology. The spatial scale of the first oxidative event and the spatial scale of the tissue response are distinct questions [1,6,34].

This distinction is especially relevant to mechanistic claims based on single time points. An early signal of mitochondrial depolarization may document an intracellular stress response, while a later reduction in tumor perfusion may reflect vascular dysfunction, edema, platelet activation, or thrombosis. Both can be true without proving that one fully accounts for tumor control. Longitudinal sampling and compartment-specific endpoints are therefore more informative than assigning a single mechanism from a single molecular marker.

Preclinical studies support a temporal separation between immediate photochemical injury, early changes in perfusion, and later inflammatory or immune-cell responses. The precise order, magnitude, and timing vary with the PS, vascular context, tumor model, and sampling method. Accordingly, a single post-treatment biopsy or imaging time point cannot reconstruct the full sequence or establish which route was the principal cause of tumor control [4,6,35].

### 3.4. Formulation, Aggregation, and Pharmacokinetic Exposure Are Mechanistic Variables

Formulation is often discussed under the broad heading of “targeting”, but its mechanistic importance extends beyond selective tumor delivery. Free PS, liposomal or polymer-associated PS, covalent conjugates, and inorganic or supramolecular carriers can differ in aggregation, plasma-protein binding, residence time, clearance, tissue penetration, endocytosis, and organellar routing. Each of these changes can modify the concentration of photoactive PS in malignant cells, endothelial cells, macrophages, and normal tissue at the time of illumination. A formulation may increase tumor-associated fluorescence while leaving the fraction of photoactive, correctly localized PS uncertain. Conversely, a clinically useful formulation may improve safety or optical monitoring without increasing intrinsic cellular phototoxicity [27,28,29].

Aggregation is frequently insufficiently characterized under tissue-relevant conditions. It can alter absorption, excited-state behavior, reactive oxygen species (ROS) production, fluorescence, and cellular uptake. The consequence is that nominal PS mass is not necessarily equivalent to active monomeric PS concentration. For formulations designed to disassemble in a tumor microenvironment or after endocytosis, characterization should include the relevant biological medium rather than only buffer conditions. Likewise, a ligand-directed platform should distinguish target binding from internalization, endosomal escape, and final subcellular distribution. These details are not peripheral formulation data; they determine the initial injury compartment on which subsequent mechanism claims depend [27,29,36].

Carrier design adds a second layer of complexity. Nanoparticles, metal–organic frameworks, and other delivery systems can change PS solubility, plasma distribution, cellular uptake, and imaging behavior simultaneously. Their value should be judged by the exposure state they create in vivo and by the safety trade-offs that accompany it, not by nominal loading or tumor fluorescence alone [37,38,39,40,41].

Recent light-sensitive nanoparticle studies illustrate both the opportunity and the interpretive challenge of advanced PS delivery: altered optical activation, delivery efficiency, and intracellular exposure can improve response simultaneously, making it essential to measure the intermediate exposure state rather than attribute benefit to one platform feature alone [42].

### 3.5. Evidential Requirements for Context-Dependent Mechanism Attribution

The framework is most useful when applied to explicit causal questions rather than used as a post hoc labeling scheme. A persuasive study should document the candidate conditioning parameter, measure the intervening exposure state, and then assess a compartment-specific response. For example, a DLI comparison becomes mechanistically informative when altered blood-versus-tissue PS distribution is demonstrated and linked to perfusion, endothelial injury, or intracellular damage; a fluence-rate comparison is more informative when oxygen kinetics or a reacted-dose surrogate accompanies outcome data. Final tumor volume alone cannot distinguish these routes [7,8,14,15].

Mechanism assignment also benefits from deliberate contrast experiments. Chemical or genetic perturbation of a death pathway, pharmacologic modulation of vascular response, immune-cell depletion in an appropriate model, and time-resolved imaging can each strengthen attribution, while recognizing that such interventions may themselves alter the treatment context. In clinical studies, causal perturbation is often infeasible. There, cellular, vascular, and immune observations are best reported as associations with a defined exposure state unless the design supports stronger causal inference. This preserves cross-protocol comparability across PSs, tumors, and anatomical sites [6,9,43].

## 4. Cellular Mechanisms: From Organellar Stress to Regulated Cell Death

### 4.1. Direct Cellular Injury Is Heterogeneous Rather than Synonymous with Apoptosis

PDT can trigger apoptosis, necrosis or necrosis-like lysis, autophagy-associated responses, ferroptosis-related lipid peroxidation, and other regulated or mixed cell-death phenotypes. The observed phenotype is influenced by PS localization, photochemical dose, cell lineage, metabolic state, redox capacity, and the timing and assays used to classify death. Consequently, a death-pathway label should be treated as a testable conclusion supported by appropriate morphology, kinetics, pathway perturbation, and functional rescue experiments—not inferred solely from one marker or one inhibitor [6,34,35,43,44].

Mitochondrial localization can disrupt membrane potential and activate intrinsic apoptotic signaling. ER-localized injury can initiate unfolded-protein and calcium-stress responses, conditions that can promote immunogenic cell death (ICD) under appropriate redox contexts, while lysosomal photodamage can release cathepsins and alter mitochondrial signaling. Plasma membrane injury can cause rapid loss of integrity and may be associated with inflammatory forms of death at higher effective doses. These examples are useful mechanistic heuristics, but no organelle should be mapped deterministically to one outcome. Cellular trafficking and stress adaptation can redirect the downstream response, and multiple compartments are commonly affected in the same treatment [6,26,34,43,45,46].

Autophagy illustrates the importance of functional interpretation. After PDT, autophagic flux may be cytoprotective, may accompany irreversible injury, or may contribute to cell death depending on the model and intervention. The presence of microtubule-associated protein 1 light chain 3 (LC3)-associated changes alone cannot resolve these alternatives. Similar caution applies to oxidative stress markers, caspase cleavage, lipid peroxidation, and inflammatory cytokines. A clinically meaningful mechanism claim requires a link between the marker, a perturbation experiment where feasible, and the outcome of interest [6,34,35,45].

### 4.2. Lipid Peroxidation and Ferroptosis-like Phenotypes After PDT

Available evidence for ferroptosis after PDT is heterogeneous, model-specific, and insufficient to support ferroptosis as a general mechanism across photosensitizers or tumor types. Canonical ferroptosis is an iron-dependent form of regulated cell death characterized by lethal lipid peroxidation; pathway assignment is strengthened by convergent lipid-peroxidation and iron-related measurements together with rescue by radical-trapping antioxidants and/or iron chelation [44,47]. Accordingly, PDT-induced lipid oxidation should be described as a ferroptosis-like or ferroptosis-associated phenotype unless pathway-specific testing distinguishes it from overlapping oxidative, apoptotic, lysosomal, and membrane-disruptive responses.

Shui et al. reported that hypericin-PDT initiated a distinct ferroptosis-like pathway through non-enzymatic lipid peroxidation that was not dependent on classical ferroptosis executors [44]. This finding supports lipid peroxidation as a relevant PDT outcome in that model; it does not establish canonical ferroptosis as a universal PDT mechanism.

Additional primary studies illustrate the model dependence and methodological heterogeneity of this phenotype. Photosens-PDT in GL261 cells showed mixed apoptotic and ferroptosis-associated components that were attenuated by ferrostatin-1 and deferoxamine [48]. In cholangiocarcinoma models, Yan et al. linked PDT to concurrent ferroptosis- and apoptosis-associated changes involving the p53/GPX4/SLC7A11 axis, whereas Dong et al. reported that hematoporphyrin-mediated PDT combined with erastin or lenvatinib increased oxidative and lipid-peroxidation-related readouts while suppressing antioxidant defenses [49,50]. Hypericin-PDT was separately linked to GPX4 suppression through AKT/mTORC1 signaling [51]. In evidentiary terms, the pathway-discriminating experiments reported by Shui et al. [44] provide the clearest support in this set for a distinct ferroptosis-like process; the rescue-based mixed-pathway findings in [48] are supportive but not definitive; and the pathway-associated or combination-treatment studies [49,50,51] should be interpreted more cautiously unless iron dependence, lipid-radical rescue, and exclusion of competing death pathways converge.

Importantly, Sleptsova et al. recently extended PDT–ferroptosis analysis to seven primary cultures derived from high-grade human gliomas. Porphyrazine III-based PDT produced culture-dependent mixed regulated cell-death profiles with a prominent ferroptotic component in several cultures, alongside apoptotic and/or necroptotic contributions; photosensitizer subcellular distribution also varied among the primary cultures [52]. This study provides patient-derived evidence beyond established cell lines while still lacking the intact perfusion, systemic pharmacokinetics, and host immunity of an in vivo tumor setting.

The relative contribution of lipid peroxidation after PDT is likely to depend on membrane localization of the PS, cellular redox buffering, iron handling, oxygen availability, and competing stress responses. Reduced viability, lipid-peroxidation markers, or changes in GPX4/SLC7A11 alone are insufficient to establish canonical ferroptosis when apoptosis or non-specific membrane oxidation has not been excluded. Rescue by ferrostatin-1 or deferoxamine strengthens the inference but is not independently diagnostic in the high-ROS setting of PDT, because radical trapping and iron chelation can also modify broader oxidative chemistry. The current evidence remains comparatively limited and model-specific; ferroptosis should therefore not be treated as a default PDT mechanism. Studies should use convergent rescue, iron-dependence, lipid-peroxidation, and pathway-relevant measurements and should state explicitly whether the findings support canonical ferroptosis or a ferroptosis-like phenotype [44,47,48,49,50,51].

Despite these limitations, ferroptosis-oriented PDT strategies remain mechanistically relevant because PDT can initiate membrane lipid oxidation and, in selected models, combine with perturbation of iron handling or the SLC7A11–glutathione–GPX4 defense system to increase oxidative cell death [44,49,50,51]. This may provide a complementary route of tumor-cell killing when conventional apoptotic readouts do not account for the response; however, the advantage is not generalizable across photosensitizers or tumor types and may be constrained by oxygen availability, membrane localization, redox buffering, iron metabolism, and normal-tissue lipid oxidation. Most mechanistic evidence still derives from established cell lines and murine tumor models [44,48,49,50,51], although the recent primary-human-glioma study provides an initial patient-derived bridge [52]. Further validations in patient-derived organoids, ex vivo tumor specimens, and patient-derived xenografts are still needed before clinical generalization. In such models, rescue by lipid-radical trapping and iron chelation should be combined with direct lipid-peroxidation measurements and exclusion of competing death pathways [44,47,48,49,50,51].

### 4.3. Redox State, Metabolism, and Resistance Modify Cellular Response

Tumor cells are not passive photochemical substrates. Antioxidant capacity, glutathione metabolism, iron handling, mitochondrial reserve, hypoxia signaling, autophagic competence, PS efflux, and repair pathways can all modify PDT sensitivity. These features may vary within the same lesion and may be altered by prior systemic therapy. Resistance should therefore be analyzed as a mechanistic category rather than invoked as a residual explanation after a negative result. Measuring only bulk viability can obscure whether failure reflects insufficient intratumoral PS, inadequate light penetration, oxygen limitation, vascular mismatch, or cell-intrinsic stress adaptation [16,53,54].

Strategies intended to overcome cellular redox buffering or hypoxia are biologically plausible but address different constraints. In this cellular context, the relevant questions are whether an intervention changes antioxidant capacity, glutathione metabolism, iron handling, mitochondrial reserve, or stress adaptation. Improvements in an in vitro oxygen-sensitive readout do not by themselves demonstrate improved tumor oxygenation in vivo, and oxygen-modifying carriers may change biodistribution and oxygen supply. These effects should be separated with direct measurements where possible [16,53,54,55].

Hypoxia-oriented strategies are mechanistically heterogeneous. Perfluorocarbon carriers, catalytic oxygen-generating materials, radical-generating photosensitizers, and oxygen-economizing formulations address different constraints and should not be treated as interchangeable interventions [55,56,57,58]. Their proposed oxygen effects should be distinguished from platform-mediated delivery effects and, where feasible, evaluated using direct oxygen measurements or reacted-dose surrogates. Dynamic oxygen depletion, resupply, and fractionation are considered separately in Section 6.2.

Within this framework, tumor–host context is treated as a modifier of exposure and response rather than as a comprehensive molecular taxonomy of PDT susceptibility. Baseline hypoxia and perfusion influence oxygen resupply; stromal architecture can affect PS penetration and light distribution; and immune competence, inflammatory state, and prior treatment may alter damage processing and adaptive amplification. Consequently, the same nominal regimen can generate different exposure states and downstream responses across lesions. These variables should be measured when central to the hypothesis rather than invoked retrospectively to explain discordant outcomes [4,9,16,53,54].

## 5. Vascular Mechanisms: An Active and Protocol-Sensitive Component

### 5.1. Endothelial Injury, Permeability, and Occlusion

Vascular response is not merely a secondary consequence of tumor-cell death. Depending on the intravascular or endothelial availability of the PS, vascular-targeted PDT can produce endothelial injury, vasoconstriction, platelet activation, increased permeability, leukocyte adhesion, flow reduction, and, in appropriate circumstances, sustained vascular occlusion. These processes may deprive the lesion of oxygen and nutrients, intensify indirect tumor injury, and alter subsequent drug delivery. The magnitude and durability of vascular effects depend on the PS, DLI, light conditions, vessel maturity, tissue architecture, and host hemostatic response [3,5,32,59,60].

Vascular injury also entails trade-offs. Increased permeability may facilitate immune-cell entry or combination-drug delivery in selected contexts, but it can also promote edema, impair local function, or create heterogeneous perfusion. Rapid shutdown may enhance local ischemic damage while limiting later delivery of systemically administered agents. Thus, the desirable vascular endpoint depends on treatment intent and sequence. A manuscript should not equate any reduction in perfusion with an optimal therapeutic state without considering timing, surrounding tissue risk, and compatibility with planned combination therapy [3,5,59,60].

Clinical VTP illustrates the value of this distinction. VTP regimens have been developed with deliberately short drug–light intervals and vascularly active PSs in selected urologic contexts. Their rationale and safety considerations differ from cell-targeted PDT protocols. Such clinical examples do not establish that vascular injury is universally dominant in PDT, but they demonstrate that the vascular compartment can be intentionally exploited as a central therapeutic target [32,33].

The CLIN1001 PCM301 randomized phase III trial provides a comparative efficacy and safety context for padeliporfin VTP in selected low-risk localized prostate cancer. It does not, however, apportion vascular, cellular, and immune contributions or establish a universal VTP mechanism [61].

Outside localized prostate cancer, phase I experience with WST-11 VTP in upper-tract urothelial carcinoma has established feasibility, safety, and local response assessment in a distinct endoluminal setting, but it does not directly quantify the relative contribution or durability of vascular occlusion [33]. More broadly, vascular injury can contribute to other PDT regimens, yet the term VTP should be reserved for protocols designed to target the vascular compartment and supported by evidence of the intended vascular exposure and sustained dysfunction or occlusion, rather than inferred from a transient post-treatment decrease in flow alone [3,5,59,60].

Preclinical Tookad-VTP imaging has directly documented rapid occlusion of tumor feeding and draining vessels in a mouse model. This supports vascular injury as an experimentally measurable route of action, but it cannot by itself establish clinical predominance in other PSs, tumor sites, or illumination geometries [62].

### 5.2. Treatment Sequence and Temporal Coordination of Mechanisms

Mechanistic predominance can evolve during and after a single illumination. An early phase may include PS excitation, oxygen consumption, and immediate injury in cellular or vascular targets. Minutes to hours later, perfusion changes, inflammation, and the release of intracellular content can reshape the treated microenvironment. Days later, residual tumor-cell survival, stromal remodeling, antigen presentation, and host immune responses may influence durable control. The sequence is not fixed, but its temporal nature cautions against conclusions based on a single post-treatment interval [4,6,7].

Temporal sequence is mechanistically relevant for combination regimens. If an immune-modulating treatment depends on antigen presentation or immune-cell access, concurrent vascular shutdown could have a different effect from delayed shutdown. If a cytotoxic drug depends on lesion perfusion, the order relative to vascular-targeted PDT becomes experimentally important. Combination claims should consequently specify the temporal rationale and measure whether the proposed enabling event actually occurred. Apparent synergy in tumor volume alone cannot identify the mechanism of interaction.

Vascular–immune crosstalk can reshape the microenvironment in which inflammatory and immune signaling occurs. Ischemic injury, perfusion loss, and cell death may alter damage-associated molecular pattern (DAMP) release, antigen availability, and immune-cell access. Whether these changes favor productive antigen presentation, ineffective inflammation, or local immunosuppression depends on the treatment context and host microenvironment. These relationships should therefore be presented as testable, coupled hypotheses rather than as a fixed vascular-ischemia-to-immune sequence [4,9,45,63,64].

## 6. Light Delivery, Oxygen Dynamics, and Photodetection

### 6.1. Incident Light Is Not Intratumoral Light

Tissue optical properties, treatment geometry, source calibration, fiber position, contact conditions, and motion all influence the fluence distribution that reaches the target. In hollow organs, interstitial treatments, surface lesions, and deep tissue, the relationship between prescribed and intratissue light can differ substantially. This problem is not only technical. Underexposure may masquerade as biological resistance, whereas superficial overexposure may exaggerate apparent activity in an accessible compartment while leaving deeper disease insufficiently treated [8,14,65].

Fluence rate is particularly consequential because it shapes the balance between photochemical consumption of oxygen and oxygen replenishment from perfusion and diffusion. Lower fluence rates have in some settings preserved oxygenation and improved response compared with higher rates delivering the same total fluence, although the direction and magnitude of the effect are model- and regimen-dependent. This literature supports a general methodological principle rather than a universal prescription: report fluence rate and evaluate oxygen-sensitive endpoints when oxygen availability is central to the hypothesis [7,8,14,15,66,67,68].

Original murine studies illustrate why this principle must not be converted into a universal low-fluence-rate rule. In one Photofrin model, lower fluence rate was associated with oxygen recovery and longer tumor-regrowth delay; in a separate study, pairing a higher fluence rate with a lower PS dose conserved oxygenation and maintained response. These regimen-specific findings support measurement of oxygen and exposure state rather than a fixed dosing prescription [67,68].

### 6.2. Fractionation Is a Biological Intervention

Interrupting illumination can allow partial reoxygenation, alter PS photobleaching, and change the time course of vascular and cellular injury. Fractionation is therefore not merely a convenient light-delivery technique. Its value depends on the kinetics of oxygen recovery, PS persistence, vascular response, and the total treatment duration. The same fractionation schedule may be helpful in one setting and neutral or detrimental in another. Studies should specify dark intervals, the rationale for their duration, and any direct evidence of reoxygenation or changed photochemical output [7,8,14].

Recent work on dermatologic fractionation further supports treating interrupted illumination as a biological intervention rather than a purely operational choice. However, fractionation results from superficial dermatologic protocols should not be transferred directly to deep oncologic or interstitial PDT without considering PS pharmacokinetics, tissue optics, and perfusion [69].

Because hypoxia can be present before treatment and can be induced during illumination, oxygen should not be treated as a binary variable. Baseline hypoxia, cycling perfusion, and treatment-induced oxygen consumption are distinct phenomena. A strategy that supplies oxygen may address one limitation but not another, and an oxygen-independent radical pathway may still remain constrained by PS delivery and light penetration. Clear mechanistic language prevents overgeneralization from a single oxygen-related intervention [16,53,54].

Recent analyses of oxygen limitation and singlet-oxygen chemistry reinforce that baseline hypoxia, dynamic oxygen depletion, and oxygen resupply are distinct experimental problems. Oxygen-enhancing platforms, including photoacoustic nanodroplets and oxygen-economizing liposomes, remain model-specific interventions whose impact on intratumoral oxygenation and clinical benefit should be directly measured where feasible rather than inferred from formulation design [58,70,71,72,73].

### 6.3. Mechanism-Linked Photodetection and Dosimetry

Photodetection can move PDT studies beyond nominal dose reporting, but different modalities interrogate different components of the exposure state. Useful measurements include PS fluorescence or lifetime, photobleaching, reflectance, intratissue fluence, oxygen- or perfusion-sensitive signals, treatment-field imaging, and singlet-oxygen-related optical signals. No single surrogate is universally sufficient; selection should follow the exposure state or mechanism under study [8,14,15,65,74].

PS fluorescence and fluorescence lifetime primarily inform localization, concentration-related signal, and microenvironmental behavior, but are affected by attenuation, self-quenching, and formulation-dependent photophysics. Reflectance helps characterize absorption and scattering; photobleaching provides a time-resolved exposure surrogate; intratissue fluence measures light delivery; and oxygen or perfusion measurements assess whether illumination is operating in an oxygen- or flow-limited state. Singlet-oxygen luminescence is closer to the intended photochemical event but remains technically demanding and spatially constrained [8,14,15,65,74].

In practice, these modalities occupy different feasibility tiers. Surface fluorescence or reflectance can be incorporated into accessible-lesion workflows but remains sensitive to attenuation, geometry, and calibration. Interstitial fluence, oxygen, or spectroscopic measurements provide localized information in deep targets but add invasiveness, fiber-placement error, and device-specific calibration burdens. Perfusion imaging is more widely interpretable for vascular response but is indirect and strongly dependent on acquisition timing, whereas singlet-oxygen luminescence remains a specialized, low-signal research measurement. Universal numerical thresholds for depth, signal-to-noise ratio, or cost are not defensible across devices, wavelengths, and tissues; feasibility should therefore be reported for the intended anatomy and decision rather than assumed from technical availability [8,14,15,65,74,75,76].

Clinical PpIX fluorescence and dual-wavelength monitoring demonstrate quantitative feasibility in selected superficial settings but do not resolve optical heterogeneity in deep lesions [75,76]. The measurement should be matched to the proposed mechanism: PS signal and fluence for cell-oriented exposure, perfusion imaging for vascular response, and local exposure plus cell-death and immune-cell dynamics for immune-oriented claims. Mechanism-linked dosimetry is therefore more informative than technology-centered monitoring.

### 6.4. Quantitative Characterization of the Biologically Active Exposure State

Current quantitative practice is component-based. Explicit and reacted-dose dosimetry are among the most developed approaches for characterizing photochemical exposure because they integrate selected combinations of PS signal or concentration, light, oxygen, and time-dependent reaction measurements. Vascular and immune responses require additional measurements and are better reported as separate dimensions of the exposure–response profile rather than folded into a single mechanistic score [8,13,14,15,77,78,79].

Quantitative characterization should distinguish at least four domains when they are relevant to the study question: (i) PS-associated exposure, assessed with calibrated fluorescence, lifetime measurements, or tissue pharmacokinetics while accounting for attenuation and self-quenching; (ii) optical exposure, assessed with measured or modeled intratissue fluence and fluence rate; (iii) oxygen dynamics, assessed with baseline and time-resolved oxygen-sensitive measurements; and (iv) vascular conditions, assessed with perfusion or vascular-function measurements when vascular exposure or shutdown is central. PS compartmental distribution and vascular perfusion are related but non-equivalent measurements and should not be used interchangeably [8,14,15,65,74,75,76].

In patients, measurement depth and invasiveness should be matched to anatomy. Surface optical measurements may be feasible in accessible lesions, interstitial probes may be justified in selected deep or intracavitary treatments, and singlet-oxygen luminescence remains a specialized research measurement. Results should be reported as a context-specific exposure profile with calibration, spatial sampling, timing, and uncertainty rather than collapsed into an unvalidated score. Where only one or two components can be measured, they should be prespecified according to the mechanism and clinical decision under study [8,13,14,15,65,74,75,76,77,78,79].

### 6.5. From Moni0074oring Signals to Clinically Actionable Dosimetry

For clinical translation, a monitoring signal is potentially actionable when it can identify underexposure, impending normal-tissue injury, inadequate field coverage, or a parameter that could be adapted during treatment. Demonstrating that a signal changes after illumination is insufficient; its relationship to the relevant exposure state and outcome must be established [8,14,15].

Prospective validation should test whether a monitoring metric improves prediction beyond conventional treatment parameters. Appropriate outcomes may include acute tissue response, perfusion change, local control, toxicity, or a prespecified molecular endpoint, depending on treatment intent. Validation should specify the intended decision, measurement uncertainty, spatial sampling limitations, calibration methods, and the temporal relationship between the signal and illumination. A metric that predicts response in a superficial homogeneous model should not automatically be extrapolated to deep, heterogeneous, or moving targets.

Early proof-of-concept theranostic platforms—including gold nanorod–PS complexes, photosensitizer-conjugated carbon dots, gold vesicles, and upconversion nanoparticles—demonstrated the technical feasibility of integrating optical tracking with phototherapy [80,81,82,83]. Their historical relevance is the establishment of integrated imaging-and-treatment concepts, not validation of contemporary clinical dosimetry. More generally, fluorescence visibility is not equivalent to calibrated photochemical exposure; imaging-guided response claims should be validated against intratissue exposure and clinically relevant outcomes, especially when a nanostructured system simultaneously alters delivery, optics, and oxygen handling.

### 6.6. Recent Clinical and Translational Dosimetry Evidence

Recent work strengthens the case for mechanism-linked dosimetry while also illustrating its setting dependence. Reviews of personalized tumor dosimetry and current dosimetry challenges emphasize that fluence, PS signal, tissue optical properties, oxygenation, and photochemical reaction metrics are complementary rather than interchangeable measures [13,77].

Clinical and translational studies in pleural Photofrin-mediated PDT have evaluated dose–response relationships and reactive-oxygen-species explicit dosimetry in a defined treatment geometry [78,79]. These studies are important proof-of-principle for exposure-informed PDT, but they do not establish a single universal dose metric across PSs, tumor sites, or treatment geometries. Their main relevance to the present framework is that a mechanistic interpretation becomes more credible when operational dosimetry is linked prospectively to an appropriate biological or clinical endpoint.

## 7. Immune Modulation: Promise, Evidence Boundaries, and Testable Claims

### 7.1. Local Inflammation Is Not Synonymous with Systemic Antitumor Immunity

PDT can provoke local inflammatory signaling, recruitment of innate immune cells, release or exposure of damage-associated molecular patterns, and changes in antigen availability. Under some conditions, these events can support adaptive antitumor responses. This biological rationale has motivated combinations with checkpoint blockade, vaccines, immune agonists, and delivery platforms designed to favor ER stress or ICD-associated signals. Yet these steps form an evidence hierarchy, not a single endpoint. Demonstrating calreticulin exposure, adenosine triphosphate (ATP) release, high-mobility group box 1 (HMGB1) release, dendritic-cell maturation, tumor infiltration, abscopal delay, and durable clinical benefit requires progressively stronger and different evidence [4,9,45,63,84].

The immune consequences of PDT may also be modified by treatment context outside the PS-light-oxygen triad. Recent work examining intraoperative PDT reported that surgical inflammation altered the ensuing immune response, underscoring the need to account for timing, tissue injury, and concomitant interventions when interpreting immune readouts [85].

A recent study in two immunocompetent murine cutaneous squamous cell carcinoma models reported that vitamin D priming combined with ALA-PDT increased ICD-associated markers, intratumoral innate and CD8+ immune-cell recruitment, and interferon-related signaling [86]. Because the intervention combined a priming agent with PDT and remained entirely preclinical, it supports context-dependent immune modulation in those models rather than clinical systemic immune efficacy or a universal immune effect of ALA-PDT.

The term ICD should be used carefully. ICD is not established by the presence of one damage-associated marker alone. Consensus criteria emphasize the need for functional evidence and appropriate interpretation in the relevant experimental context. In PDT research, ER stress and ICD-associated biomarker profiles can be highly informative, particularly when a PS is designed to access the ER, but they remain insufficient by themselves to establish a therapeutic vaccine effect in patients [9,45,63,84].

The same PS can yield divergent immune consequences because PS identity does not fix the exposure state. DLI can shift the initial injury toward vascular or cellular compartments; fluence rate and fractionation alter oxygen depletion and tissue stress; vascular shutdown changes antigen release and immune-cell access; and treatment sequence determines whether immune modulation occurs before or after these events. Baseline immune infiltration, prior therapy, stromal architecture, and immunoregulatory networks further shape whether PDT-associated inflammation becomes productive adaptive immunity, ineffective inflammation, or local immunosuppression. Divergent findings with the same PS should therefore prompt comparison of protocol and tumor–host context rather than be treated as inherently contradictory [3,4,5,7,8,9,14,15,16,45,63,64].

Direct serial assessment of DAMP release in patients is difficult because repeated tumor sampling at defined intervals after illumination is invasive and often impractical. In clinical protocols with planned resection or biopsy, paired tissue may permit analysis of ICD-associated markers, cell-death context, and local immune-cell changes. When serial tissue is not feasible, peripheral immune profiling, circulating inflammatory markers, imaging of inflammatory or immune-cell changes, and limited on-treatment biopsies may provide complementary information. These measures are supportive or exploratory: none alone establishes tumor-cell ICD, functional systemic immunity, or causal clinical benefit. Human studies should therefore prespecify sampling time, comparator, assay purpose, and the clinical outcome to which the immune readout will be linked [9,11,12,45,63,64,84,87].

Clinical evidence for PDT-induced systemic immunity remains more limited and heterogeneous than the preclinical literature. Small studies, non-uniform regimens, variable immune assays, and concomitant therapies make causal attribution difficult. Review-level syntheses and trial-watch articles are useful maps of this literature, but they aggregate heterogeneous reports and should not be treated as independent proof of clinical immune efficacy [11,12,87]. This limitation should not be read as evidence against immunologic relevance; rather, it identifies the need for prospective studies with prespecified immune endpoints, adequate temporal sampling, and clinically meaningful outcomes. Claims should be proportionate to the level of evidence: ‘ICD-associated changes’ is often more accurate than ‘PDT induces durable systemic immunity’ in early-stage work [4,9,11,12,64,87].

**Figure 1:**
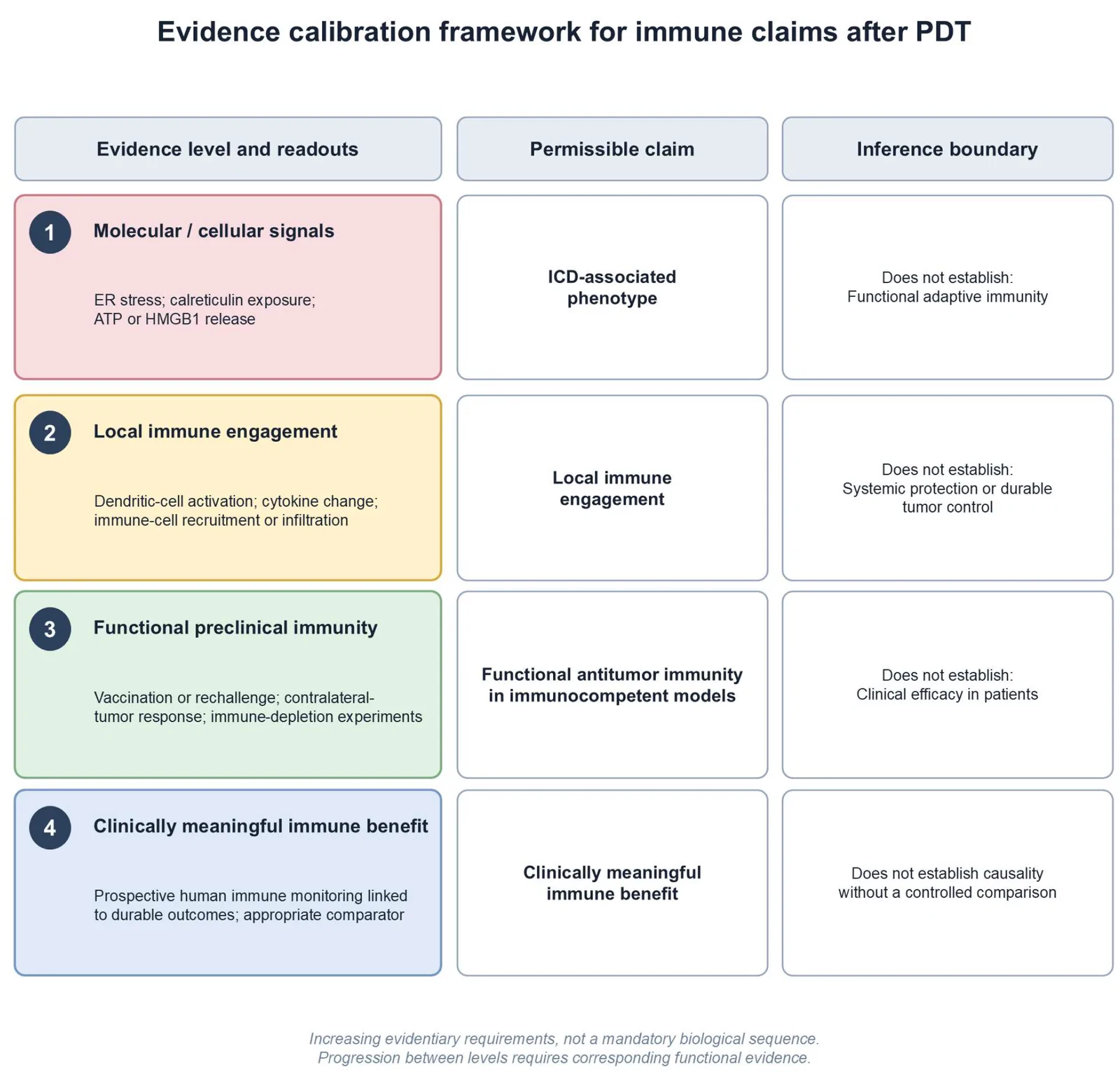
Evidence calibration framework for immune claims after PDT. Each level lists representative readouts, the claim that the evidence can support, and the conclusion that it cannot establish. The four levels indicate increasing evidentiary requirements rather than a mandatory biological sequence; progression between levels should not be inferred without corresponding functional evidence. ICD, immunogenic cell death; ER, endoplasmic reticulum; ATP, adenosine triphosphate; HMGB1, high-mobility group box 1. Figure is created for this review. Colors are used only to distinguish different evidence levels, and arrows indicate increasing evidentiary requirements rather than a mandatory biological progression.

Figure 1 summarizes representative readouts, permissible claims, and inference boundaries across increasing levels of immune evidence; Table 1 translates these levels into practical minimum evidence requirements and residual limitations.

### 7.2. Minimum Experimental Evidence Required for Immune Claims

Table 1 should be used as a claim-calibration tool rather than as a checklist. Immune-oriented studies should document treatment exposure, characterize the relevant death context, use functional experiments where feasible, distinguish local from systemic endpoints, and report negative or mixed results. In clinical studies, DAMP-related, blood-based, or imaging measurements should be interpreted as supportive evidence unless they are linked to functional immune assays and relevant clinical outcomes. Across current preclinical and early translational studies, evidence strength varies with the PS, tumor model, immunological endpoint, and use of rechallenge, vaccination, or other functional tests; clinical reports generally do not provide randomized evidence of systemic immune efficacy. These observations should therefore be described as regimen-specific preclinical or early translational evidence rather than established clinical immune benefit [9,11,12,45,63,64].

## 8. From Parameters Through Exposure State to Relative Mechanism Contributions

The framework proposed here is intentionally a matrix rather than a hierarchy. A parameter can influence more than one mechanism, and a mechanism can be shaped by several parameters simultaneously. The table below is not a dosing guide. It is a structured prompt for study design and manuscript interpretation: identify the parameter, measure an appropriate intermediate endpoint, and avoid claiming a dominant mechanism without evidence proportionate to the claim.

The matrix also highlights why “dominant mechanism” should be a comparative, evidence-based statement. A tumor-volume response after PDT is compatible with direct cytotoxicity, vascular compromise, immune contribution, or a mixture. To infer relative predominance, investigators need measurements that discriminate among these routes and, where possible, perturbation experiments. In clinical studies, direct perturbation may be infeasible; in that case, mechanistic language should remain associative and be framed as a biologically plausible interpretation rather than proof. For immune-oriented interpretations, the level of claim should additionally be calibrated against the hierarchy summarized in Figure 1 and Table 1.

### 8.1. Discordant Findings Are Expected and Should Be Explained

Different studies can reach apparently conflicting conclusions about a PDT mechanism without either result being incorrect. A PS that is largely cell-associated in one model may remain vascularly available in another; a fluence rate that preserves oxygenation in one geometry may not do so in a lesion with different perfusion; and an inflammatory response can be productive or ineffective depending on immune context and treatment sequence. The appropriate response to such discordance is not to force a universal hierarchy, but to report the exposure state and identify which component differs between protocols [8,14,15,16,53].

**Figure 2:**
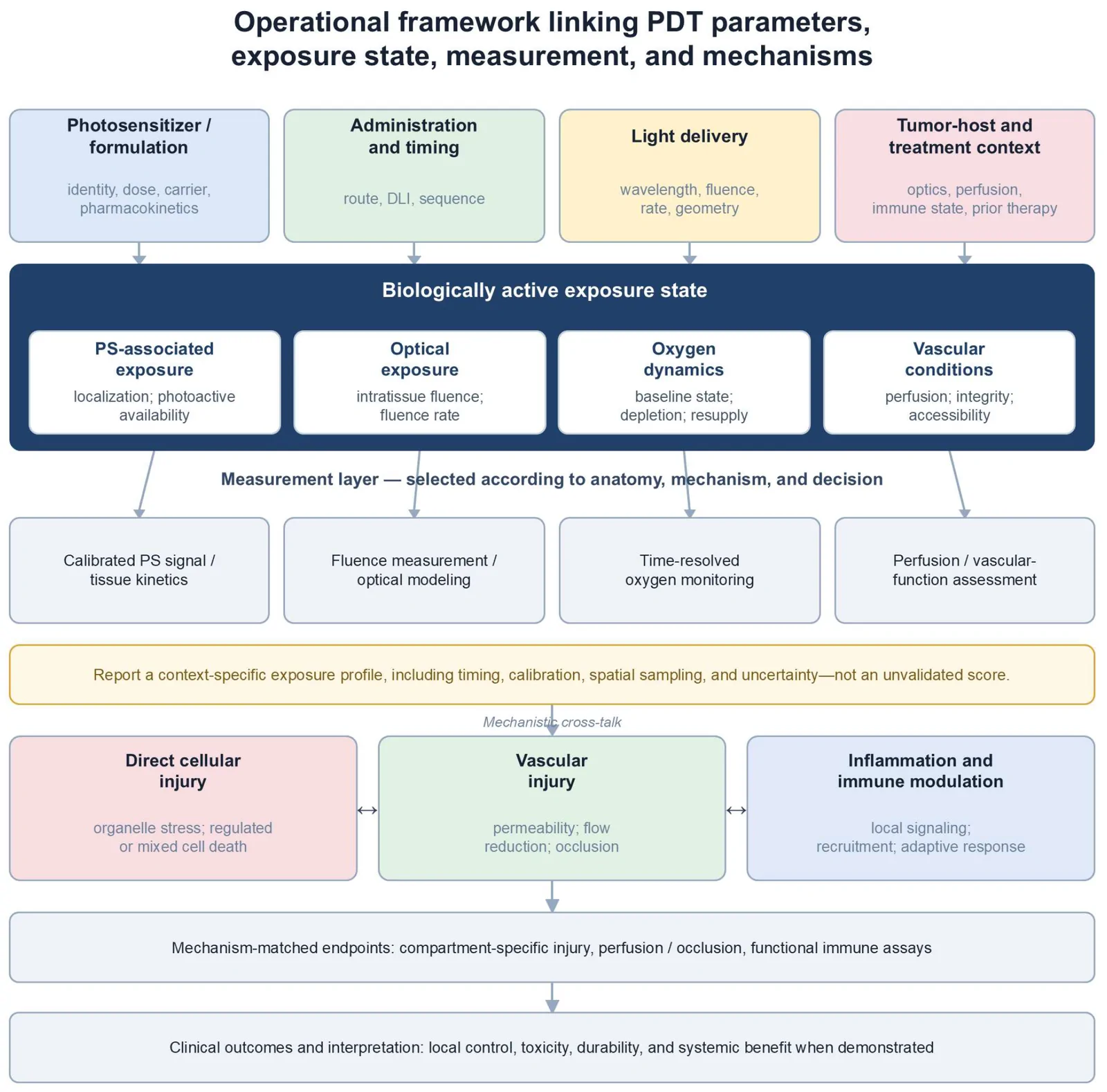
Operational parameter–exposure state–measurement–mechanism framework for oncologic PDT. Protocol inputs and baseline tumor–host contexts shape a multidimensional exposure state. Selected measurements characterize PS-associated exposure, intratissue light delivery, oxygen dynamics, and vascular conditions before mechanism-matched biological endpoints are interpreted. The exposure state should be reported as a context-specific profile rather than a universal composite score. The framework is intended for study design and claim calibration, not for clinical dosing. PDT, photodynamic therapy; PS, photosensitizer; DLI, drug–light interval. Figure is created for this review. Colors are used for visual distinction of conceptual domains, and arrows indicate the directional relationship between parameters, exposure states, measurements, and mechanistic interpretation rather than a fixed biological sequence.

Figure 2 operationalizes this logic by placing a measurement layer between the biologically active exposure state and mechanistic interpretation. The intermediate state comprises PS-associated exposure, the intratissue light field, dynamic oxygen availability, and vascular conditions; each component is linked to a measurement approach selected for the anatomy and study question. The resulting context-specific profile then constrains claims about direct cellular injury, vascular injury, or immune modulation. Figure 2 presents the operational architecture of the framework, whereas Table 2 provides parameter-specific consequences and discriminating endpoints. The figure is a study-design framework, not a deterministic algorithm or clinical dosing score.

### 8.2. Illustrative Applications of the Framework

Consider a comparison in which the PS, dose, illumination protocol, and tumor model are held constant while DLI is varied. The input change is timing, but the mechanistically relevant intermediate is the blood-versus-tissue distribution state at illumination. A framework-based study would therefore measure PS kinetics or localization and pair these data with perfusion or endothelial endpoints and compartment-specific cellular injury. A difference in tumor control alone would not identify whether the relative vascular or cellular contribution had changed [7].

Likewise, identical total fluence delivered at different fluence rates can generate different oxygen trajectories. The relevant exposure state includes intratissue fluence, oxygen availability, and photochemical consumption; informative endpoints may therefore include oxygen or reacted-dose surrogates, early perfusion changes, and tumor response. Regimen-specific findings showing benefit from a lower fluence rate in one setting but preserved response with a higher rate and lower PS dose in another illustrate why the framework favors measurement of the intervening exposure state over a universal dosing rule [14,15,67,68].

These examples show the incremental function of the framework: it does not merely relabel mechanisms after an outcome is observed but specifies which intermediate measurements and contrast endpoints are needed to distinguish competing explanations.

## 9. Translational Implications

### 9.1. Match the Intervention, Endpoints, and Clinical Claim

PDT is best described as a biologically active treatment system, not solely as a drug plus a device. Clinical reports should describe the protocol sufficiently to reconstruct the intended exposure state. When targeted formulations are used, target identity, evidence of engagement, and pharmacokinetic consequences should be reported separately from therapeutic response [1,3,30].

Endpoint selection should follow the intended mechanism. Studies designed to examine direct cell injury should include compartment-appropriate cellular endpoints and avoid overinterpreting non-specific viability changes. Vascular-targeted studies should assess perfusion or vessel function over time. Immune-oriented studies should incorporate an evidence hierarchy that includes functional immune endpoints rather than only cytokine panels. In all cases, normal-tissue injury, anatomic constraints, and the feasibility of repeated illumination need to be considered alongside tumor response.

### 9.2. Combinations Require a Causal Rationale

PDT combinations are attractive because PDT can provide local cytotoxic injury while other therapies address systemic disease, immune suppression, hypoxia, or residual survival pathways. The rationale should nevertheless identify which constraint the partner treatment is expected to address. A checkpoint inhibitor cannot be assumed to convert local inflammation into durable immunity; likewise, oxygen-modifying materials do not correct inadequate light delivery, and targeted carriers do not necessarily improve every component of the therapeutic index. Combination studies are strongest when they test the proposed enabling mechanism and define treatment sequence a priori [2,88,89].

### 9.3. Clinical Context Should Determine the Mechanism Question

The most relevant mechanistic question differs across clinical applications. In an accessible superficial lesion, adequate surface fluence and normal-tissue selectivity may dominate feasibility. In an endoluminal or biliary target, light-delivery geometry, treatment-field coverage, and obstruction-related clinical endpoints become central. In an interstitial treatment, three-dimensional placement, tissue optical heterogeneity, and the relationship between planned and delivered dose require special attention. In VTP, early perfusion response and off-target vascular injury may be more informative than a tumor-cell apoptosis marker. A review that groups all of these interventions under a single PDT mechanism risks losing the clinical variables that actually constrain benefit [3,31,32,60].

Clinical translation also requires realistic attention to safety. The spatial selectivity of illumination does not eliminate toxicity: PS persistence can create photosensitivity, and local inflammation, edema, strictures, pain, or tissue necrosis may matter differently by anatomical site. A regimen designed to maximize inflammatory or vascular effects may not be appropriate near critical structures. Maximizing phototoxicity should therefore not be treated as a universally desirable objective. The therapeutic index must be interpreted in the relevant organ, treatment geometry, and standard-of-care context [3,30,32,90,91].

Mechanism-informed early-phase trials should be designed not only to assess safety and response but also to determine whether the intervention achieved the intended biological exposure state. Here, ‘feasible’ means one or two prespecified pharmacokinetic, optical, perfusion, or immune measurements selected for the treatment geometry, sampling access, calibration capacity, and decision they are intended to inform—not a broad exploratory panel. Such embedded translational endpoints can help distinguish failure of target exposure or treatment delivery from biological resistance or failure of the mechanistic premise.

### 9.4. Barriers to Clinical Translation of Novel Photosensitizers

Photophysical potency alone does not establish clinical suitability. Candidates optimized for absorption, fluorescence, singlet-oxygen yield, or cell killing in simplified systems may behave differently in biological media because aggregation, protein binding, formulation stability, pharmacokinetics, tissue penetration, clearance, and subcellular routing determine the photoactive fraction present at illumination [1,3,27,28,29,37,38,39,40,41,42]. Consequently, high tumor-associated fluorescence or in vitro reactive-oxygen-species production cannot substitute for calibrated exposure and a reproducible therapeutic window.

A second barrier is the need to develop the PS together with a clinically feasible light-delivery and monitoring strategy. A compound may remain difficult to translate when the intended anatomy cannot be illuminated reproducibly, when intratumoral exposure cannot be characterized, or when prolonged photosensitivity, off-target accumulation, or local tissue injury limits the therapeutic index. Translation also requires a clearly defined clinical indication in which the regimen offers a practical benefit relative to existing care. These constraints explain why early attention to formulation, dosimetry, safety, treatment geometry, and the anatomical use case is more informative than photophysical potency alone [2,3,8,13,14,15,30,65,77,90,91].

### 9.5. Clinical Evidence Map and Inference Boundaries

The clinical PDT literature should be used according to what it can establish. Studies in cholangiocarcinoma, dermatologic oncology, prostate VTP, and upper-tract urothelial carcinoma demonstrate that PDT can be technically and clinically deployed in anatomically distinct settings; they do not, by themselves, establish a common molecular mechanism. The intended target compartment, light geometry, and clinically relevant endpoint differ substantially between these applications. Disease-specific clinical evidence should therefore be interpreted alongside, rather than substituted for, mechanistic measurements [31,32,33,61,92,93].

Recent clinical syntheses in head and neck cancer and dermatology likewise show that clinical PDT protocols differ substantially in photosensitizer, light-delivery route, treatment objective, and toxicity constraints [94,95]. These studies support the need for disease- and protocol-specific interpretation, rather than cross-indication extrapolation of a single mechanism or dose–response rule.

Table 3 illustrates how a clinical application can be mapped to an explicit mechanism question and an appropriate endpoint. The representative settings were selected to illustrate distinct treatment geometries, target compartments, and evidentiary boundaries rather than to provide a comprehensive survey or comparative efficacy ranking. The evidence levels are not interchangeable: randomized clinical data support selected disease-specific efficacy questions, whereas early-phase studies primarily establish feasibility and safety, and guideline or review evidence does not establish mechanistic causality. This mapping helps prevent a response observed in one anatomical setting from being used to support an unmeasured mechanism in another. Future prospective studies should prespecify which evidence would support or weaken the claimed cellular, vascular, or immune contribution.

### 9.6. Reporting Expectations for Clinical PDT Studies

Clinical PDT studies face an inherent tension between mechanistic ambition and trial feasibility. Mechanism-linked sampling can be valuable but should be limited to measurements that directly address a prespecified question and can be interpreted alongside the primary efficacy and safety endpoints. When key exposure parameters are incompletely reported, mechanistic conclusions become difficult to validate or compare across studies or treatment sites.

### 9.7. What Current Clinical Studies Do Not Yet Resolve

The available clinical literature has demonstrated feasibility and reported site-specific activity for selected PDT and VTP applications, but it rarely provides the serial compartment-resolved measurements required to apportion cellular, vascular, and immune contributions. This is a limitation of the evidence base rather than a failure of individual trials. Future protocols can address it by embedding a small number of mechanism-linked measures (for example, PS kinetics and light-field verification, early perfusion assessment for VTP, or prespecified local and systemic immune sampling) alongside clinically relevant efficacy and safety endpoints [32,33,60,92,93].

Importantly, such embedded studies should not overburden early-phase trials with exploratory assays that cannot be interpreted. The mechanism question, sampling time, analytical method, and decision rule should be specified in advance. This approach would make negative and positive trials informative, because an absence of benefit could then be separated into insufficient exposure, biological resistance, or a failed mechanistic premise.

Combination platforms that integrate PDT with chemotherapy, photothermal treatment, or immune-oriented delivery may be useful experimental tools, but they also make causal attribution more difficult. When multiple components change simultaneously, studies should include component controls and avoid assigning an observed benefit to PDT-driven immunity or oxygen modulation without direct evidence [96,97]. For example, the upconversion-nanoparticle plus checkpoint-blockade study represented by [96] was conducted in a murine colorectal cancer model and cannot establish clinical combination efficacy.

## 10. Recommendations for Future Studies and Reporting

The following recommendations emphasize six principles rather than a universal parameter checklist: characterize the intervention as a time-resolved system; measure an informative exposure surrogate where feasible; predefine the candidate mechanism; use discriminating, mechanism-matched endpoints; align sampling with the expected temporal sequence; and state explicitly the evidence level and uncertainty of each mechanistic claim. Detailed reporting items are consolidated in Table 4.

A practical reporting priority is to make the exposure state and mechanistic inference transparent enough for studies to be compared and later synthesized. This can be achieved without standardizing every PDT regimen. It is particularly important as targeted PS platforms and imaging-guided systems become more complex, because each added component can improve control while introducing additional sources of biological variation.

### 10.1. Comparative Experiments and Models That Can Test the Framework

The framework can be tested prospectively through controlled contrasts that vary one protocol dimension while measuring the intervening exposure state. Such studies should prespecify competing mechanistic explanations, include measurements of distribution, oxygenation, perfusion, or immune-cell kinetics as appropriate, and use endpoints capable of discriminating among cellular, vascular, and immune contributions. Factorial or sequence-sensitive designs may be particularly informative for multicomponent formulations and combination regimens, in which several determinants change simultaneously.

No single preclinical model captures the full clinical context. Two-dimensional cultures facilitate mechanistic perturbation but omit vascular flow, tissue optics, and immune architecture. Three-dimensional models can improve spatial biology but may still lack perfusion and host immunity. Immunocompetent in vivo models permit functional immune analysis but may have PS pharmacokinetics and optical dimensions that are not directly transferable to humans. Human translational studies face the opposite challenge: clinical relevance but fewer opportunities for invasive mechanistic sampling. A coherent development pathway therefore uses each model for the question it can answer and carries conclusions forward only at the corresponding evidence level.

### 10.2. Limitations of This Critical Narrative Review

This review has several scope-related limitations. As a critical narrative review, it does not estimate field-wide prevalence, pooled effect sizes, or risk of bias for individual mechanistic findings. The focused 30-study audit was descriptive rather than probability-sampled and was coded at the PubMed-abstract level; its percentages therefore apply only to the audited cross-section, and a negative code indicates that an element was not explicit in the abstract rather than absent from the full study. Cellular, vascular, and immune contributions are difficult to isolate even experimentally and are still harder to apportion in clinical studies, where sampling is constrained, and treatment protocols are heterogeneous. The PubMed-centered search was supplemented by citation chaining but did not systematically cover chemistry, optics, materials-science, and engineering databases, so studies indexed primarily outside the biomedical literature may be underrepresented. The framework is therefore best used to structure transparent hypotheses and measurements, with mechanistic conclusions calibrated to the available evidence. Regulatory status, device availability, and emerging clinical evidence may also change as the PDT literature evolves. Finally, the synthesis is restricted to oncologic PDT; non-oncologic indications have different treatment objectives, tissue contexts, and endpoints.

## 11. Conclusions

PDT involves multiple interacting mechanisms whose relative contributions are context-dependent. Its biological effects depend on more than a PS label or prescribed light dose. PS localization and DLI help define the compartment of initial injury; light delivery, fluence rate, and oxygen dynamics shape photochemical exposure; vascular architecture and treatment sequence influence indirect tissue injury; and tumor–host context shapes inflammatory and immune consequences. Direct cellular injury, vascular damage, and immune modulation are therefore best considered interacting contributors whose relative importance can be evaluated with mechanism-matched measurements where feasible.

The next priority is prospective testing of the framework. Studies can report a context-specific exposure profile and embed one or two prespecified exposure- or mechanism-linked endpoints that are feasible for the treatment geometry—for example, light-field verification with a PS signal or early perfusion assessment for VTP—alongside efficacy and safety outcomes. Controlled contrasts can then vary one protocol dimension and test whether the measured exposure state explains the result. The framework is specific to oncologic PDT and would require adaptation for non-oncologic applications. Applied in this way, it can improve cross-study comparability while keeping translational claims proportionate to the evidence.

## Figures and Tables

**Table 1:** Minimum experimental evidence required for representative immune claims after PDT. ATP, adenosine triphosphate; DAMP, damage-associated molecular pattern; HMGB1, high-mobility group box 1; ICD, immunogenic cell death. Evidence concepts are synthesized from [9,11,12,45,46,63,64].

Claim Level	Minimum Supporting Evidence	Residual Limitation
ICD-associated phenotype	Multiple DAMP-related measurements (e.g., calreticulin exposure and ATP or HMGB1 release) with appropriate controls and cell-death context	Does not establish functional adaptive antitumor immunity
Local immune engagement	Time-resolved activation or infiltration of relevant immune populations, supported by appropriate local functional assays where feasible	Does not establish systemic protection or durable tumor control
Functional preclinical immunity	Contralateral-tumor, rechallenge, vaccination, or immune-depletion experiments in immunocompetent models	Does not establish clinical efficacy in patients
Clinically meaningful immune benefit	Prospective immune monitoring linked to durable clinical outcomes, ideally with an appropriate comparator	Causal attribution remains limited without controlled comparisons

**Table 2:** Parameter–exposure state–mechanism–measurement matrix for context-dependent interpretation of PDT. PS, photosensitizer; DLI, drug–light interval. Framework is synthesized from [1,5,7,8,14,15,26].

Parameter or Context	Likely Exposure-State Consequence	Mechanistic Contributions Potentially Altered	Measurements and Discriminating Endpoints
PS identity and formulation	Changes pharmacokinetics, aggregation, tissue distribution, and localization at illumination	Cellular, endothelial, or stromal contributions	Calibrated PS signal or tissue kinetics; subcellular colocalization; distribution at illumination
DLI	Changes the circulating-versus-tissue-associated PS fraction and compartment exposure	Relative vascular or cellular contribution	Blood/tissue PS kinetics; compartmental localization; perfusion; compartment-specific damage
Fluence rate and fractionation	Changes the temporal delivery of intratissue fluence and the balance between oxygen consumption and resupply	Magnitude and spatial distribution of direct photochemical, vascular, and mixed injury	In vivo oxygen measures; photobleaching; intratissue fluence; reacted-dose surrogate
Baseline and dynamic oxygen state	Distinguishes pre-existing hypoxia, treatment-induced depletion, and oxygen resupply	Photochemical efficiency, spatial injury, and secondary vascular effects	Baseline and time-resolved oxygenation; perfusion; reacted-dose surrogate
Light geometry and tissue optics	Changes the spatial distribution of intratissue fluence	Superficial versus deep-tissue effects and normal-tissue injury	Fluence mapping; imaging; treatment-field verification
Vascular architecture	Changes perfusion reserve and endothelial targetability	Vascular shutdown, permeability, and ischemic contribution	Dynamic perfusion imaging; leakage; thrombosis markers
Tumor–host immune context	Changes inflammatory processing of photochemical and tissue injury	Local immune engagement or adaptive immune amplification	Immune-cell kinetics; functional immune assays; local and systemic immune outcomes
Treatment sequence and combination timing	Changes whether perfusion, oxygenation, antigen release, or partner-drug exposure occurs before or after vascular or cellular injury	Interactions among cellular, vascular, and immune contributions	Component controls; prespecified sequence; time-resolved exposure and mechanism-matched endpoints

**Table 3:** Representative clinical contexts and the corresponding mechanistic inferences (whether they can or cannot be supported). ALA, 5-aminolevulinic acid; CCA, cholangiocarcinoma; MAL, methyl aminolevulinate; PS, photosensitizer; VTP, vascular-targeted photodynamic therapy; WST-11, padeliporfin (TOOKAD Soluble). This is an interpretive map rather than a comparative efficacy synthesis [31,32,33,61,93,95].

Clinical Context	PS/Treatment Setting	Evidence Type	Core Clinical Endpoint	Mechanistic Inference Boundary
Superficial non-melanoma skin cancer	Topical ALA- or MAL-PDT in selected lesions	Guideline and contemporary review evidence	Lesion response and site-specific toxicity	Does not establish systemic immune benefit.
Unresectable CCA	Systemic PS plus endoluminal PDT	Randomized prospective clinical study	Survival, cholestasis-related outcomes, and treatment-related safety	Does not define an organellar or immune mechanism.
Prostate VTP	Padeliporfin (WST-11; TOOKAD Soluble) VTP	Phase III randomized trials and clinical treatment studies	Biopsy-defined progression and genitourinary safety	Clinical response does not apportion vascular, cellular, and immune contributions.
Upper-tract urothelial carcinoma VTP	Padeliporfin (WST-11; TOOKAD Soluble) VTP	Phase I study	Feasibility, safety, and local response	Early-phase activity does not establish a universal VTP mechanism.

**Table 4:** Reporting considerations for mechanism-oriented PDT studies. PS, photosensitizer; DLI, drug–light interval; ICD, immunogenic cell death. These considerations were informed by [8,14,15,88,89,96,97] and by the broader evidence synthesis presented in this review. This is not a consensus guideline.

Reporting Domain	Core Information That Improves Interpretability
Photosensitizer (PS)	Chemical identity, formulation, route, dose, DLI, and any targeting ligand or carrier
Light delivery	Wavelength, source, calibration, fluence, fluence rate, geometry, fractionation, and treated volume/area
Exposure-state characterization	PS signal or tissue kinetics; intratissue light field or surrogate; oxygen or perfusion when relevant; calibration, timing, spatial sampling, and uncertainty
Mechanistic endpoints	Compartment-specific cellular, vascular, and/or immune endpoints with clearly reported sampling times
Immune claims	ICD-associated markers distinguished from functional immune assays and clinical outcomes
Combination design	Component controls; treatment sequence, interval, and mechanistic rationale; and verification that the proposed enabling event occurred
Translation	Normal-tissue effects, anatomic feasibility, reproducibility, and compatibility with the intended clinical context

## Data Availability

The study-level coding and summary data supporting the focused descriptive content audit are provided in Supplementary Table S1. No other new datasets were generated or analyzed for this critical narrative review.

## Supplementary Materials

The following supporting information can be downloaded at: https://www.mdpi.com/article/10.3390/ijms27167465/s1.
